# Co-Expression of miRNA Targeting the Expression of PERK, but Not PKR, Enhances Cellular Immunity from an HIV-1 Env DNA Vaccine

**DOI:** 10.1371/journal.pone.0018225

**Published:** 2011-03-25

**Authors:** Adam K. Wheatley, Marit Kramski, Marina R. Alexander, Jesse G. Toe, Rob J. Center, Damian F. J. Purcell

**Affiliations:** 1 Department of Microbiology and Immunology, University of Melbourne, Melbourne, Victoria, Australia; 2 Division of Infection and Immunity, The Walter & Eliza Hall Institute of Medical Research, Melbourne, Victoria, Australia; Indian Institute of Science, India

## Abstract

Small non-coding micro-RNAs (miRNA) are important post-transcriptional regulators of mammalian gene expression that can be used to direct the knockdown of expression from targeted genes. We examined whether DNA vaccine vectors co-expressing miRNA with HIV-1 envelope (Env) antigens could influence the magnitude or quality of the immune responses to Env in mice. Human miR-155 and flanking regions from the non-protein encoding gene *mirhg155* were introduced into an artificial intron within an expression vector for HIV-1 Env gp140. Using the miR-155-expressing intron as a scaffold, we developed novel vectors for miRNA-mediated targeting of the cellular antiviral proteins PKR and PERK, which significantly down-modulated target gene expression and led to increased Env expression *in vitro*. Finally, vaccinating BALB/c mice with a DNA vaccine vector delivering miRNA targeting PERK, but not PKR, was able to augment the generation of Env-specific T-cell immunity. This study provides proof-of-concept evidence that miRNA effectors incorporated into vaccine constructs can positively influence vaccine immunogenicity. Further testing of vaccine-encoded miRNA will determine if such strategies can enhance protective efficacy from vaccines against HIV-1 for eventual human use.

## Introduction

Despite intensive research and the development of new generation vectors and delivery modalities, broadly protective vaccines against many common chronic viral infections, such as HCV and HIV-1, have met with limited clinical success. Many groups are currently focussed upon identifying strategies to improve antigen expression and/or immunogenicity, vaccine delivery and efficacy. One potential area for improvement of vaccination strategies using recombinant viral vectors and/or pure nucleic acid for the expression of viral antigens may lie in preventing cellular antiviral responses that limit efficient antigen expression.

In mammalian cells, multiple and overlapping intracellular antiviral response pathways mediate the detection of viral infection and the induction of early innate immune effectors. Productive infection results in the accumulation of viral components, for example, double-stranded RNA (dsRNA) or virion structural proteins, which are recognised by host surveillance proteins such as interferon inducible, dsRNA-dependent protein kinase R (PKR, EIF2AK2) [1] and PKR-like ER kinase (PERK, EIF2AK3) [2]. PKR can be activated via intracellular signalling in response to Type 1 interferons [3], or by direct binding of dsRNA [4] and upon activation, PKR mediates multiple functions including the phosphorylation of eukaryotic initiation factor 2-α (eIF-2α) [5], the activation of transcription factors IκB and NFκB [6] and the induction of apoptosis by interactions with pro-apoptotic mediators such as Fas-associated death domain (FADD) [7] or C/EBP homologous protein (CHOP) [8]. eIF-2α is an essential factor required for the initiation of mammalian mRNA translation [9] and the phosphorylation of eIF-2α prevents recycling back into the ribosomal initiation complex leading to a cell-wide shutoff of protein synthesis [10]. The activity of PKR can be positively and negatively regulated by interactions with cellular proteins such as PKR-activating protein (PACT, PRKRA) [11], [12] or TAR-RNA binding protein (TRBP) [13].

A parallel, cellular homeostatic pathway with antiviral activity is the unfolded protein response (UPR), or endoplasmic reticulum (ER)-stress response pathway. Expression and folding of viral or cellular glycoproteins within the ER is guided by a series of protein chaperones including the binding Ig protein (BiP) [14]. The overexpression or misfolding of proteins within the ER preferentially recruits BiP from heterodimeric complexes containing one of three cellular proteins; i) activating transcription factor 6 (ATF6), ii) inositol-requiring kinase 1 (IRE1) or iii) PERK [2]. The release of either ATF6 or IRE1 increases the transcription of UPR-specific molecular chaperones, thereby relieving the accumulated protein load [15], [16]. Upon release from BiP, PERK catalyses the phosphorylation of EIF-2α [17], with sustained translational inhibition leading to the triggering of pro-apoptotic pathways and cell death [18]. Thus, during normal cell homeostasis, the UPR regulates protein synthesis to ensure protein fidelity. However during infection, when viral proteins are over-expressed to favour copious production of virions, the UPR enforces a limit on expression and induces apoptosis to slow viral replication and spread.

Unsurprisingly many common viruses have evolved mechanisms to circumvent the activation of innate antiviral pathways. For example, E3L protein of vaccinia virus (VACV) [19] or the TRS1 protein of cytomegalovirus [20] inhibit the activation of PKR by binding and sequestering viral dsRNA. In addition, many viruses simultaneously inhibit the UPR, for example, hepatitis-C virus (HCV) E2 protein can bind and sequester PERK [21]. Alternatively, ICP34.5 from HSV can direct cellular dephosphatase enzymes to reverse the phosphorylation of EIF-2α to allow the re-initiation of protein synthesis [22]. Although the activation and modulation of antiviral responses during viral infection is well characterised, less is known about their impact in the context of vaccination against viral pathogens and in particular, the extent to which innate antiviral surveillance may limit the optimal expression and/or the immunogenicity of the HIV-1 envelope protein (Env), a common candidate immunogen for an HIV-1 vaccine. Previously, the co-expression of the E3L and K3L proteins from VACV was shown to limit the PKR response and apoptosis resulting in increased antigen expression *in vitro* from a recombinant canarypox vector encoding HIV-1 Gag and Env [23]. Similarly, co-expression of myxoma virus M11L protein inhibits apoptosis and augments Env gp140 antigen expression from a DNA vector *in vitro*, and promotes increased Env-specific CD8+ T-cell immunity in vaccinated mice [24]. These studies suggest that preventing the activation of intracellular antiviral response pathways and/or apoptosis may allow increased Env expression *in vivo* and facilitate the induction of enhanced immune responses.

One potential mechanism to limit cellular antiviral responses is the knockdown of cellular genes by RNA interference (RNAi). The intracellular production of short 21–23 bp dsRNA duplexes, termed micro-RNAs (miRNAs), or synthetic analogues such as small interfering RNAs (siRNAs) or short hairpin RNAs (shRNAs), can mediate the post-transcriptional control of gene expression and sequence-specific gene silencing (reviewed in [25]). In previous studies, PKR-specific siRNA were utilised to prevent a PKR response following flavivirus [26] or HIV-1 infection [27]. Furthermore, the stable knockdown of PKR expression in HeLa cells using shRNA prevents EIF-2α phosphorylation and translational shutdown after treatment with the dsRNA analogue polyI:C. [28]. Similarly, knockdown of PERK expression using siRNA prevents EIF-2α phosphorylation in response to cellular stress [29], [30], confirming that reductions in the steady state expression levels of PKR and PERK can modulate the potency of intracellular antiviral responses.

In the present study, we designed and constructed DNA vaccine vectors for the co-expression of HIV-1 Env gp140 antigens and engineered miRNA targeting cellular antiviral proteins. Sequence-specific knockdown of human and murine PKR and PERK mRNA and protein levels resulted in increased Env gp140 expression *in vitro* from a fluorescent reporter. When used to vaccinate BALB/c mice, an Env gp140 DNA vaccine delivering miRNA targeting PERK, but not PKR, significantly augmented the magnitude of the Env-specific CD8+ T-cell response.

## Materials and Methods

### Oligonucleotides and PCR

Oligonucleotides were synthesised by Proligo/Sigma-Aldrich and are listed in Table 1. PCR reactions were performed using Phusion polymerase (Finnzymes) with the amplification conditions: 2 min at 95°C, 35 cycles of 10 s at 95°C, 30 s at 50°C–60°C, 15 s per kb of target sequence at 72°C, and a final extension of 7 min at 72°C.

**Table 1:** Oligonucleotides.

Name	Sequence (5′ to 3′)
Odp.M13F	gttttcccagtcacgacgttgta
Odp.70	actattgctattattattgctactac
Odp.181	cataggagatgccagtcgccgc
Odp.1212	ggtatggctgattatgatctagagtcg
Odp.1213	tatgctagcacggtgagcaagggcgaggagctgttc
Odp.1572	gtggcacaaaccagaagggg
Odp.1573	cctgcaattaagaatgacatgaatat
Odp.1582	tccgtcgcataagaagccaaggg
Odp.1583	tcccgtaataagcttgaggtgtgg
Odp.1584	ccttggcttcttatgcgacggagtggcacaaaccaggaaggggaaatctgtgg
Odp.1585	ccacacctcaagcttattacgggacctgcaattaagaatgacatgaatatattttctg
Odp.1586	cccttggcttcttatgcgacggagagtgctgaaggcttgctgtaggctgtatg
Odp.1587	ccacacctcaagcttattacgggactagtaacaggcatcatacactgttaatgc
Odp.1694	ccctgccagttggaggcaaaagcagggagtagtacttaaatacagcatacagcctacagcaagccttcagcactcag
Odp.1695	ctccctgcttttgcctccaactggcagggagtagtacttaaatacagtgtatgatgcctgttactagcattcac
Odp.1719	cacgtcagttggaggcaaaaacgtgacacgttcggagaatttacagcatacagcctacagcaagccttcag
Odp.1720	tcacgtttttgcctccaactgacgtgacacgttcggagaatttacagtgtatgatgcctgttactagcattcacatg
Odp.1721	ctctcgtttccagttggaggcaaaagaaacgagagccggatttattcagcatacagcctacagcaagccttcagcac
Odp.1722	gctctcgtttcttttgcctccaactggaaacgagagccggatttattcagtgtatgatgcctgttactagcattcac
Odp.1723	cccactgtcctcagttggaggcaaaaaggacagtgggaccttcagttcagcatacagcctacagcaagccttcagca
Odp.1724	ccactgtcctttttgcctccaactgaggacagtgggaccttcagttcagtgtatgatgcctgttactagcattcaca
Odp.1725	gtatatactccaccagttggaggcaaaagtggagtatatacactcgaaacagcatacagcctacagcaagccttcagca
Odp.1726	gtatatactccacttttgcctccaactggtggagtatatacactcgaaacagtgtatgatgcctgttactagcattcaca
Odp.1727	gatgtctgtggcttcagttggaggcaaaaaagccacagacatcattgaaacagcatacagcctacagcaagccttcagc
Odp.1728	gtctgtggcttttttgcctccaactgaagccacagacatcattgaaacagtgtatgatgcctgttactagcattcac
Odp.1729	gcctgcagatccagttggaggcaaaagatctgcaggcttgaatcattcagcatacagcctacagcaagccttcagca
Odp.1730	gcctgcagatcttttgcctccaactggatctgcaggcttgaatcattcagtgtatgatgcctgttactagcattcac
Odp.1923	catggcctccaaggagtaag
Odp.1924	tgtgaggaggggagattcag
Odp.2003	gaccagtcaacaggggacat
Odp.2004	cctgaccaaggaaagcaaag
Odp.2015	agcaggctgagcgatatgat
Odp.2016	tctcagcaccttccgtcttt
Odp.2122	gcagccaaattagctgttga
Odp.2123	cacactgttcataatttacag
Odp.2124	tgagaagtggaatttcagtgtt
Odp.2125	tgttcttccacatctgaaata
Odp.2126	agagtaaccgttggtgacata
Odp.2127	tcaggaactgcaaagcaaatact

### Plasmids

The plasmids pCMV-EGFP (formerly pEGFP), pAD8-160, pNL-160, pAD8-140 [31], pCMV-Empty [32], pcDNA3-PKR and pcDNA3-PKR-N [33] have been previously described. pNL-140, expressing Env gp140 from strain NL4.3, was created by introducing a stop codon immediately prior to the transmembrane domain and mutating the gp120/gp41 cleavage motif within pNL-160. The EGFP coding sequence with AUG start codon mutated was amplified by PCR with Odp.1212/Odp.1213 and pEGFP-N1 (Clontech) and fused in-frame with *env* gp140 to create the pNL-140.EGFP reporter.

DNA sequences from the human miRNA-155 host gene (*mir155hg*) (GeneID:114614) were amplified by PCR with primers Odp.1572/Odp.1573 and cDNA generated from human PBMC and cloned into vector pCMV-Empty to create the plasmid pMIR155HG. Using pMIR155HG as a template, a 112 bp or 446 bp PCR fragment encompassing the miR-155 primary micro-RNA (pri-miRNA) and flanking regions was amplified using primers Odp.1586/Odp.1587 and Odp.1584/Odp.1585 respectively. Two additional PCR reactions using pAD8-140 as a template were used to amplify the 5′ and 3′ regions of an artificial intron, bounded by the CMV intron A splice donor (SD) and C57Bl/6 IgG heavy chain splice acceptor (SA), using primer pairs Odp.M13F/Odp.1582 and Odp.1583/Odp.181 respectively. The 5′ intron, 3′ intron and miR-155 containing PCR products were joined together by PCR using primers Odp.M13F/Odp181 and sub-cloned into the pAD8-140 vector using NcoI and NotI. Computer based RNA folding analysis using mFold 3.0 [34] indicated that the stem loop structure of the pri-miRNA was preserved.

Mature miRNA (Table 2) modelled on a non-silencing (NS) siRNA (Qiagen) (Odp.1719/Odp.1720) or designed to target mRNA for huPKR (GeneID:5610) (Odp.1694/Odp.1695), huPERK (GeneID:9451) (Odp.1721/Odp.1722), huPACT (GeneID:8575) (Odp.1723/Odp.1724), muPKR (GeneID:19106) (Odp.1725/Odp.1726), muPERK (GeneID:13666) (Odp.1727/Odp.1728) or muPACT (GeneID:23992) (Odp.1729/Odp.1730) were generated by overlap extension PCR using the indicated primer sets and cloned into pNL-140 and pNL-140.EGFP using NcoI and NotI. The authenticity of all DNA segments created by PCR was confirmed by DNA sequencing.

**Table 2:** Engineered miRNA sequences.

Target	Mature miRNA Sequence (5′ – 3′)
Non-silencing control	**UGUACUCCAGCUUGUGCCCTT**
Human PKR	**UAUUUAAGUACUACUCCCUGC**
Human PERK	**AAUAAAUCCGGCUCUCGUUUC**
Human PACT	**AACUGAAGGUCCCACUGUCCU**
Murine PKR	**UUUCGAGUGUAUAUACUCCAC**
Murine PERK	**UUUCAAUGAUGUCUGUGGCUU**
Murine PACT	**AAUGAUUCAAGCCUGCAGAUC**

### Cell culture and Transfection

HeLa human cervical epithelial cells and LTA murine fibroblast cells [35] were maintained at 37°C, 5% CO_2_ in DMEM (Invitrogen/Gibco BRL) supplemented with 10% (v/v) heat inactivated FCS (JRH Biosciences) and 100 U/ml streptomycin and penicillin. Cells were seeded in Nunc 6-well plates (ThermoFisher) at 3.5–4×10^5^ cells/well and transfected with the respective plasmids the next day using Lipofectamine™ 2000 (Invitrogen) according to the manufacturer's instructions. Total DNA amounts per transfection were kept constant by supplying the vector backbone pCMV-Empty or pcDNA3, and where appropriate transfection efficiency was equalised by co-transfection of 0.25 µg of pDS-Red-N1 (Clontech).

### Flow Cytometry

Forty-eight hours post transfection, cells were detached with trypsin, washed and resuspended in PBS+50 mM EDTA. Events were collected using a FACSCalibur cell sorter (BD) and analysed for EGFP, DS-Red and 7-AAD fluorescence using Weasel v2.5 software (Walter & Eliza Hall Institute, Melbourne, Australia). Cells were gated based upon DS-Red expression to control for relative transfection efficiency and cell viability was assessed by the cellular exclusion of 7-AAD (BD).The relative fluorescence intensity of Env.EGFP expression was determined as the product of the proportion and mean fluorescent intensity of fluorescent cells.

### SDS-PAGE and Western blot

Adherent cells were detached 48 h post-transfection using PBS+50 mM EDTA, washed in PBS and lysed in 80 µl of TTS buffer (10 Mm Tris pH 8.0, 0.5% (v/v) Triton-X 100, 150 mM NaCl, 2 mM PMSF, 1 µg/ml aprotonin, 1 µg/ml leupeptin). Cells were incubated for 15–30 min on ice before insoluble material was pelleted by centrifugation at 10000 rpm, 10 min, 4°C. The total protein concentration of cell lysates were measured and equalised using a Pierce BCA Protein Assay Kit (Thermo Scientific), diluted 1∶5 with SDS loading buffer and resolved by 8% SDS-PAGE. Resolved proteins were transferred to Polyscreen PVDF membrane and probed using antibodies to HIV-1 gp120 Env (D7324, Aalto Bioreagents), EGFP (632375, Clontech), PKR (F9, [36]), PERK (ab31373: Abcam), PACT (ab31967, Abcam) or β-actin (A5060, Sigma-Aldrich) and detected using horseradish-peroxidase (HRP)-conjugated secondary antibodies (Zymed) and Pierce Supersignal West Pico Chemiluminescent substrate (Thermo Scientific). Bands were visualised and densitometry performed using Kodak 4000MM Image Station and IM software (Carestream Health).

### Denaturing Urea-PAGE and Northern Blot

Total cellular RNA was extracted from cells using Trizol reagent (Invitrogen) and 10 µg of RNA was resolved by 7M Urea/15% denaturing-PAGE as previously described [37]. Membranes were probed with either a miR-155 specific (5′-acccctatcacgattagcattaa-3′) DNA oligonucleotide probe end-labelled with γ-^32^P dATP, or a U6 snRNA specific PCR probe with incorporated α-^32^P dCTP [38], washed and exposed to a Fujifilm BAS-MS-IP 2340 plate for 48 h. Blots were visualised using a FLA3000 phosphoimager and Image Gauge software (Fujifilm Corp).

### RT-qPCR

10 µg of total cellular RNA was treated for 3 h at 37°C with RQ1 DNase (Promega) before heat inactivation at 65°C for 10 min. cDNA was generated from 1 µg of purified, DNase treated RNA using random hexamer primers and AMV reverse transcriptase (Promega) according to the manufacturer's protocol. A no RT control using water instead of the reverse transcriptase was included to ensure complete DNA digestion. cDNA together with the indicated primer pairing and Stratagene Brilliant II Fast SYBR-Green qPCR master mix were used for real-time quantitative PCR (RT-qPCR) amplification of huPKR (Odp.2122/Odp.2123), huPERK (Odp.2124/Odp.2125) and huPACT (Odp.2126/Odp.2127) according to the manufacturer's instructions. Duplicate experimental reactions, no RT and no template controls were run using the Mx3000P (Stratagene) instrument. On each plate, three reference gene amplicons were also amplified using the indicated primers: huGAPDH (GeneID:2597) (Odp.1923/Odp.1924), huYWHAZ (GeneID:7534) (Odp.2015/Odp.2016) and huHPRT1 (GeneID:3251) (Odp.2004/Odp.2005). The geometric mean of the relative expression of the reference genes was calculated using a previously published formula [39] and used to normalise the expression for the cellular genes of interest.

### Enzyme-linked immunosorbant assay (ELISA)

The level of soluble Env in the culture supernatants of transfected cells was determined by capture ELISA using the anti-Env sheep antibody D7324 as previously described [31]. Culture supernatants were serially diluted in block buffer and added for 2–4 h to allow Env gp140 capture and incubated with a 1∶1000 dilution of pooled HIV-1 positive patient sera in block buffer. After 2 h incubation, the primary antibody was detected using HRP-conjugated anti-human IgG antibody (Dako) and 3,3-5,5-tetra-methyl-benzidine (TMB) substrate at 450 nm.

### Animal Immunisations

All studies involving the use of animals were approved by the University of Melbourne animal ethics committee (Ethics approval number: 06190). Groups of female 6–8 week old BALB/c mice were immunised with a total of 100 µg of plasmid DNA diluted in 100 µl of PBS via intramuscular injection into both hind gastrocnemius muscles. Injections were given under ketamine/xylazine-induced anaesthesia on three occasions, one week apart, followed by the collection of whole spleens and blood samples two weeks after the final injection.

### Intracellular Cytokine Secretion (ICS) Assay

Splenocytes were isolated from immunised mice by passage through a 70 µM filter and centrifugation using Ficoll-Paque to remove red blood cells. After washing twice with PBS, splenocytes were plated at 1×10^6^ cells/well in round-bottomed 96-well plates (Nunc) and stimulated by the addition of 1 µM/ml of the immune-dominant, H-2Dd-restricted peptide RIQRGPGRAFVTIGK (p18) [40] for 2 h at 37°C, 5% C0_2_. For the measurement of CD4+ T-cell responses, cells were re-stimulated for 12 h with 1 µg/ml purified recombinant NL4.3 Env gp140 protein [41]. Stimulated splenocytes were treated with 2 µg/ml of brefeldin-A (Sigma-Aldrich) for 5 h before the cells were re-suspended, washed with FACS Wash (PBS+5% FCS+0.1% sodium azide) and stained with antibodies to muCD4 (Clone L3T4, BD) and muCD8β (Clone H35-17.2, BD) for 30 min at 4°C. Cells were then fixed in 4% paraformaldehyde, permeablised using PERM-2 buffer (BD) and stained with rat anti-muIFN-γ (Clone XMG1.2, BD) for 45 min at 4°C. Samples were washed, fixed in PBS/1% formaldehyde and analysed using an LSRII flow cytometer (BD) and Weasel v2.5 software.

### Statistical Analyses

Experimental *in vivo* results for CD4+ and CD8+ T-cells were analysed by Mann-Whitney U tests using Graphpad Prism v4.0 software. Differences were deemed significant at a p-value<0.05. All *in vitro* experiments were analysed as mean +/− SEM and where applicable by Mann-Whitney U tests (specifically cited in text).

## Results

### The activation of PKR limits HIV-1 Env expression *in vitro*

**Figure 1:**
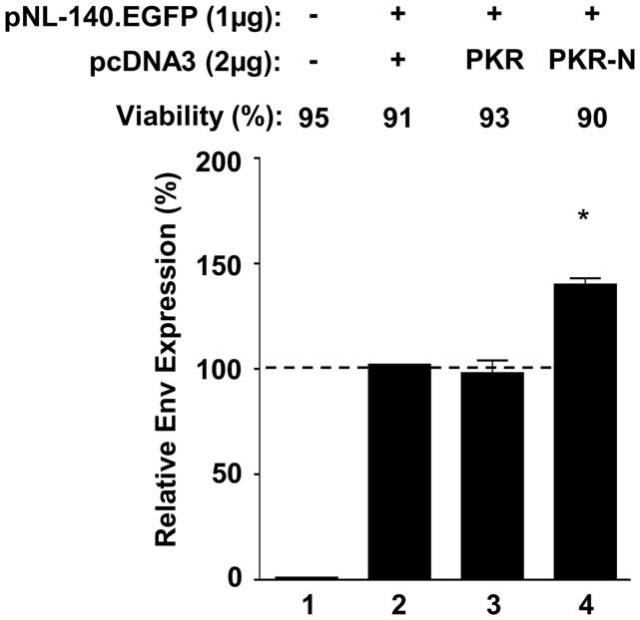
A dominant-negative PKR mutant increases Env.EGFP reporter expression. HeLa cells were analysed by flow cytometry 48 h post-transfection with the pNL-140.EGFP reporter and plasmids expressing either wild-type PKR, an N-terminal PKR mutant (PKR-N) or plasmid backbone alone (pcDNA3). Values represent the relative fluorescence of Env.EGFP normalised to reporter+backbone control alone and is presented as the Mean +/− SEM of four independent transfections. Env expression in the presence of PKR-N was compared to PKR alone using a Mann-Whitney U test. (* indicates p<0.05).

We have previously demonstrated that an activated PKR response restricts both the expression of HIV-1 structural proteins and viral replication within astrocytes and 293T fibroblasts and that the co-expression of PKR inhibitors such as TRBP, or targeting of PKR expression with siRNA, was able to rescue efficient virion production from the proviral pNL4.3 plasmid [27]. To examine if the PKR response similarly limits the efficient expression of Env from DNA vaccines, we co-transfected HeLa cells with a fluorescent HIV-1 Env reporter plasmid, pNL-140.EGFP, encoding a truncated Env gp140 fused to EGFP (Env.EGFP), together with cDNA expression plasmids expressing human wild-type PKR or the N-terminal dsRNA binding domain of PKR (PKR-N), previously shown to inhibit PKR activation in a dominant-negative fashion [42]. Enhanced Env expression (40%), as measured by increased intracellular Env.EGFP fluorescence, was observed after co-transfection with PKR-N (Figure 1). In contrast, the over-expression of wild-type PKR did not diminish Env expression compared to cells transfected with the vector backbone alone. These observations suggest that Env expression alone, in the absence of HIV-1 viral replication, may trigger intracellular antiviral surveillance which acts to partially restrict the maximal expression of Env gp140.

### Expression of correctly processed miR-155 does not inhibit Env expression

**Figure 2:**
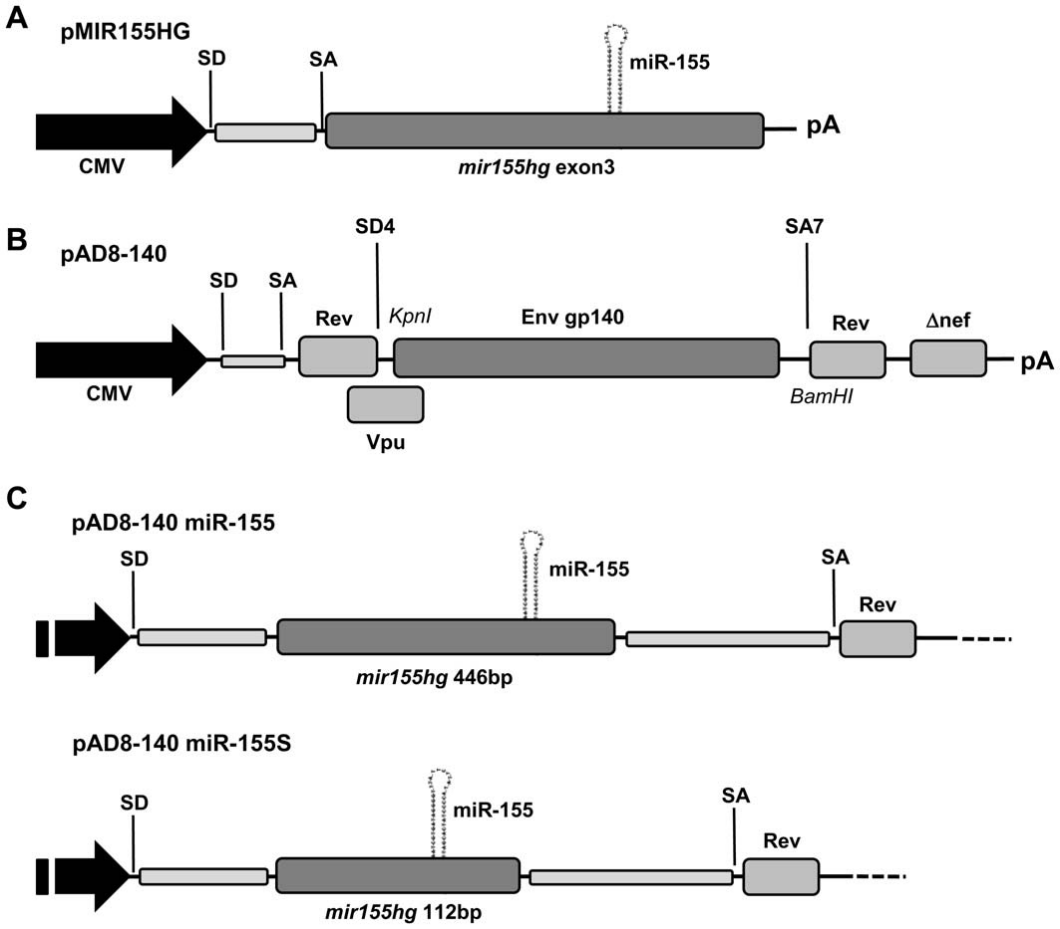
Design of Env expression plasmids for the co-expression of miR-155. (**A**) Diagram of the pMIR155HG construct. A CMV immediate early promoter drives transcription through an artificial intron consisting of the CMV intron A splice donor (SD) and C57Bl/6 IgG heavy chain splice acceptor (SA). The transcript encodes the 3^rd^ exon of the human *mir155hg* gene and terminates at a bovine growth hormone poly-adenylation signal (pA). (**B**) The HIV-1 Env expression plasmid pAD8-140, expresses a truncated and cleavage site modified Env protein (gp140) and is based on a native *env* cDNA derived after splicing of exons 1 and 4bE from the full genomic mRNA. The HIV-1 5′ UTR, *rev* and 3′ UTR regions are derived from strain NL4.3, whilst the area bounded by KpnI and BamHI encode a heterologous *env* gp140 gene from strain ADA. A CMV promoter drives transcription through the artificial intron described above. The 4 kb transcript encodes for Vpu, gp140 and a truncated Nef protein. The HIV-1 intron is bounded by SD4 and SA7 and Rev is expressed from the spliced 2 kb mRNA. (**C**) Overlap extension PCR was used to incorporate the entire MIR155HG exon 3 sequence, or a shortened 122 bp variant into the artificial intron of pAD8-140 to create the vectors pAD8-140 miR-155 and pAD8-140 miR-155S respectively.

In order to examine the importance of cellular antiviral responses during vaccination, we developed HIV-1 Env expressing DNA vaccine vectors that co-express miRNA. Sequences flanking the well characterised micro-RNA-155 (miR-155) [43], [44] were cloned behind a CMV promoter to create pMIR155HG (Figure 2A). In addition, a complete (446 bp), or truncated (112 bp) variant of the miR-155 expression cassette was inserted within an artificial intron in the DNA vaccine vector pAD8-140 (Figure 2B) [31], creating pAD8-140 miR-155 and pAD8-140 miR-155S respectively (Figure 2C), and thereby placing miRNA and Env transcription under the control of the same RNA Polymerase II (PolII)-dependent, CMV promoter. Constitutive splicing of the artificial intron was previously confirmed [32].

**Figure 3:**
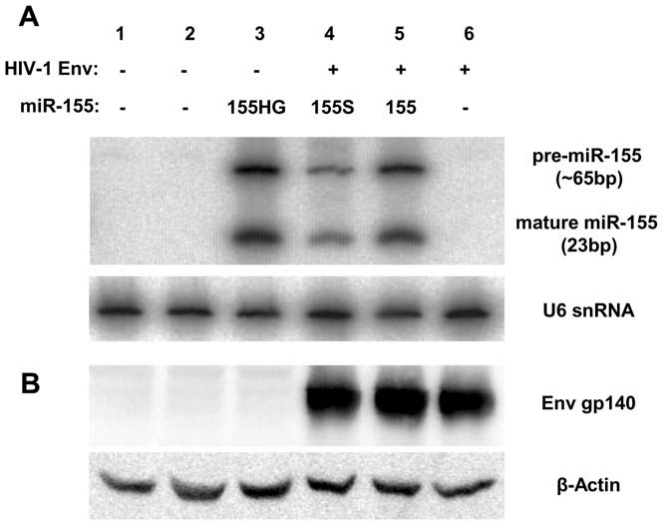
Expression of mature miR-155 does not inhibit efficient Env expression. (**A**) Un-transfected HeLa cells (lane 1) or HeLa cells transfected with 2 µg of either pCMV-Empty vector backbone (lane 2), pMIR155HG (lane 3) or Env expression vectors pAD8-140 miR-155S (lane 4), pAD8-140 miR-155 (lane 5) or pAD8-140 (lane 6). Forty-eight hours post-transfection, total RNA was isolated and resolved using denaturing urea PAGE. Membranes were subsequently probed with an [^32^P]-ATP radio-labelled oligonucleotide complementary to the mature guide strand of miR-155. Equal loading of RNA was confirmed by probing with a [^32^P]-CTP labelled, DNA probe specific for U6 snRNA. (**B**) Protein lystates were isolated from similarly transfected HeLa and resolved using SDS-PAGE. Expression levels of Env were analysed by immunoblotting with anti-gp120 D7324 and anti-β-actin antibodies.

The production of correctly processed, mature miR-155 was confirmed by northern blot analysis of RNA from HeLa cells transfected with plasmids pMIR155HG, pAD8-140 miR-155S and pAD8-140 miR-155 (Figure 3A). No endogenous production of miR-155 was detected in mock-transfected cells, or cells transfected with either the control plasmid pCMV-EGFP or the Env expression plasmid pAD8-140 (lanes 1, 2 and 6). In contrast, transfection with the plasmid pMIR155HG led to the efficient production of both the ∼65 bp pre-miRNA hairpin and the mature, 23 bp miR-155 (lane 3). Similarly, when expressed from the artificial intron of the vectors pAD8-140 miR-155S and pAD8-140 miR-155, both pre- and mature miRNAs were detected. miRNA expression from the truncated expression cassette was significantly reduced compared with expression from the full length constructs (lane 4 compared to lane 5) indicating the retention of the extended flanking regions enhanced expression of both mature and processed miR-155. This suggests that miR-155 flanking sequences are important for miRNA transcription, stability and/or Drosha processing and we therefore used the complete miR-155 cassette in all subsequent constructs. Env expression, as detected by Western blot (Figure 3B), was comparable between the two miRNA co-expression vectors and the Env expression plasmid pAD8-140 as measured by densitometry relative to β-actin, indicating that production of miR-155 from intronic mRNA did not reduce translation from the *env* mRNA through Drosha-induced cleavage of the transcript prior to splicing.

### MIR155HG sequences can act as a scaffold for heterologous, engineered miRNA targeting cellular antiviral proteins

**Figure 4:**
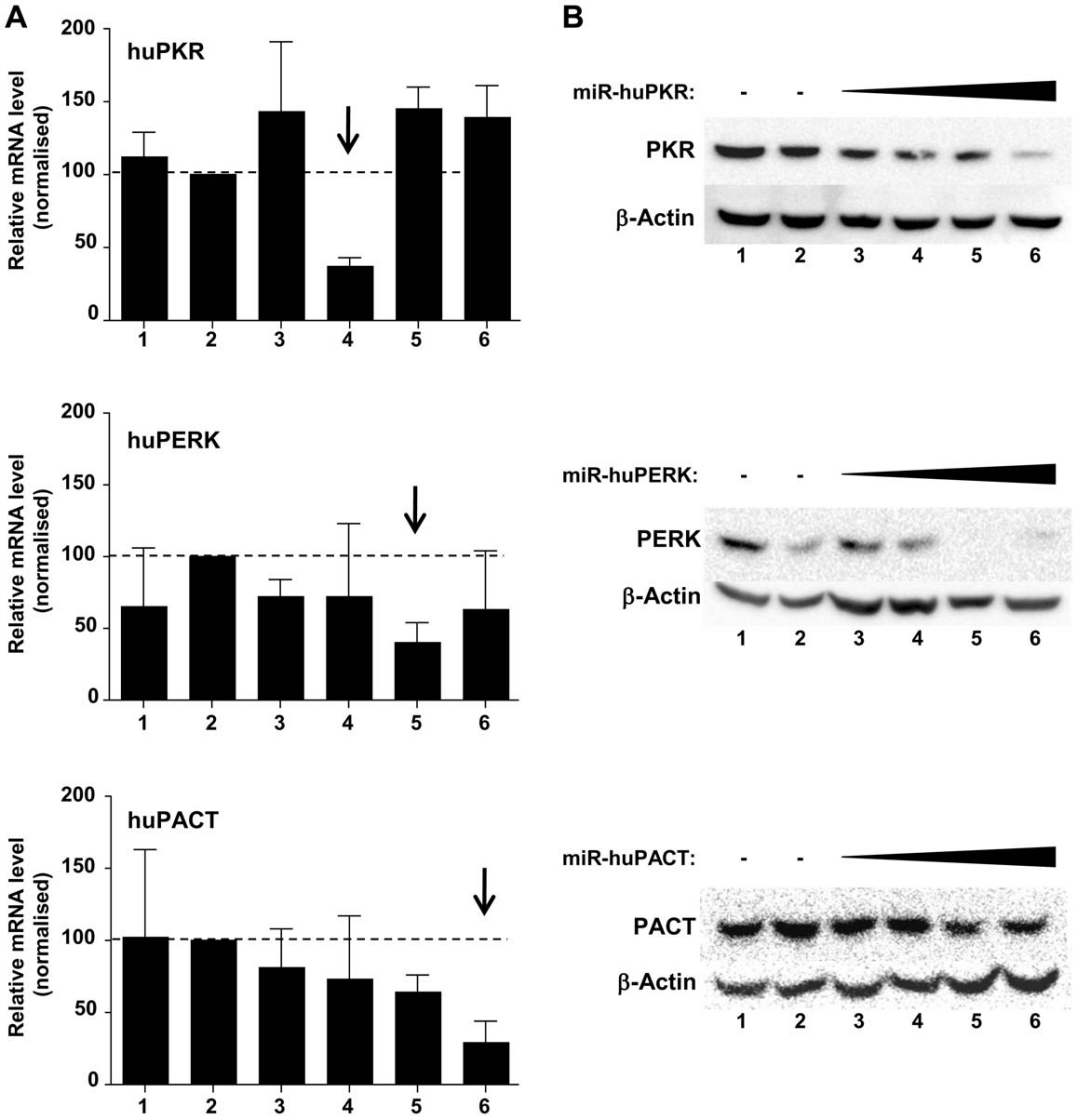
Substitution of miR-155 with an engineered miRNA directs the knockdown of cellular antiviral proteins. (**A**) RT-qPCR analysis of target mRNA knockdown. HeLa cells were transfected with 2 µg of pCMV-Empty (lane 1), pAD8-140 (lane 2), pAD8-140 miR-NS (lane 3), pAD8-140 miR-huPKR (lane 4), pAD8-140 miR-huPERK (lane 5) or pAD8-140 miR-huPACT (lane 6). Forty-eight hours post-transfection, total RNA was harvested, cDNA was generated and amplicons specific to PKR, PERK and PACT mRNAs were quantified and normalised using the relative expression of three reference genes, GAPDH, YWHAZ and HPRT1. Arrows indicate the interactions between sequence specific miRNAs and the relevant target mRNA. Results are relative to the target gene expression in cells transfected with pNL1-140 alone and is presented as the Mean +/− SEM from two independent transfections. (**B**) Western immunoblot of protein knockdown. HeLa cells were transfected with 2 µg of pCMV-Empty (lane 1), pAD8-140 (lane 2) or 0, 0.5, 1.5 and 2 µg of pAD8-140 co-expressing miRNA targeting huPKR, huPERK or huPACT (lanes 3 to 6). The total DNA concentrations were kept to 2 µg by the addition of pAD8-140 miR-NS. Cell lysates were isolated 48 h post-transfection and 75 µg of total protein was resolved using an 8% SDS-PAGE and transferred to PVDF membrane. Blots were probed with antibodies specific for human PKR, PERK, PACT or β-actin before detection with species appropriate HRP-conjugated secondary antibodies.

After confirming the efficient production of miR-155 from our Env expression vectors, we developed vectors expressing heterologous, engineered miRNAs designed to knockdown the expression of three well characterised, human antiviral proteins: huPKR, huPERK and huPACT. miRNA sequences designed to target these antiviral proteins (Table 2) were substituted for miR-155 within the intron of vector pAD8-140 miR-155. To assess the specificity of miRNA targeting, HeLa cells were transfected with plasmids expressing Env, both with and without miRNA co-expression, and the levels of target gene mRNA accumulation were measured using RT-qPCR (Figure 4A). Compared to cells transfected with the pAD8-140 alone, co-expression of miRNAs targeting huPKR, huPERK and huPACT directed the knockdown of target mRNA accumulation by ∼60–70%. mRNA levels were maximally suppressed in samples transfected with the corresponding miRNA (marked with the arrows), indicating knockdown was sequence dependant for the respective mRNA target species. Interestingly, cellular mRNA levels of huPKR were elevated after transfection with any vectors expressing miRNA, including the NS control. The expression of antiviral proteins after miRNA targeting was examined by Western immunoblot of whole cell lysates of transfected HeLa cells. Titration of increasing amounts of plasmids co-expressing miR-huPKR or miR-huPERK, relative to the NS control, resulted in a corresponding decrease in huPKR and huPERK protein expression respectively (Figure 4B). In contrast, the expression of huPACT protein remained relatively stable, despite miRNA targeting clearly leading to a depletion of huPACT mRNA 48 h after transfection.

### Knockdown of PKR and PERK but not PACT expression increases intracellular Env expression in human and murine cell lines

**Figure 5:**
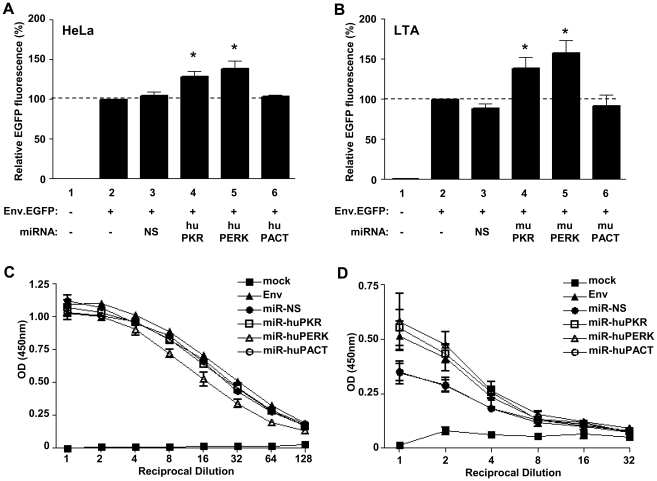
miRNA targeting murine PKR and PERK but not PACT augment Env expression *in vitro*. (**A**) Green fluorescence as a marker of Env expression from the pNL-140.EGFP reporter constructs. HeLa cells were transfected with 1 µg of pCMV-Empty (lane 1), pNL-140.EGFP (lane 2), or pNL-140.EGFP co-expressing miR-NS (lane 3), miR-huPKR (lane 4), miR-huPERK (lane 5) or miR-huPACT (lane 6). Forty-eight hours post-transfection, the relative fluorescence intensity of Env.EGFP was determined and is presented relative to cells transfected with reporter alone as the Mean +/− SEM of four independent transfections. Results were compared to the miR-NS control using a Mann-Whitney U test. (* indicates p<0.05). (**B**) LTA cells were similarly transfected with 1 µg of pCMV-Empty (lane 1), pNL-140.EGFP (lane 2), or pNL-140.EGFP co-expressing miR-NS (lane 3), miR-muPKR (lane 4), miR-muPERK (lane 5) or miR-muPACT (lane 6) and analysed for green fluorescence by flow cytometry 48 h post-transfection. The relative fluorescence intensity was determined relative to cells transfected with reporter alone and is presented as the Mean +/− SEM of four independent transfections. Results were compared to the miR-NS control using a Mann-Whitney U test. (* indicates p<0.05). Secretion of Env.EGFP from the pNL-140.EGFP reporter constructs was measured by ELISA. Half-log-2 serial dilutions of culture supernatants from (**C**) HeLa or (**D**) LTA transfected with pNL-140.EGFP alone or co-expressing miRNA were incubated in a 96-well plate coated with anti-gp120 D7324 antibodies. Bound Env.EGFP was detected using anti-HIV human sera, HRP-conjugated anti-human IgG and colorimetric reaction of the TMB substrate was measured and is shown as the Mean +/− SEM of two separate transfections.

We next examined the effect of antiviral protein knockdown upon Env.EGFP expression from the pNL-140.EGFP fluorescent reporter. The intronic expression cassettes for the engineered NS control miRNA or miRNA targeting human or murine PKR, PERK and PACT (Table 2) were cloned into pNL-140.EGFP. Plasmids were transfected into HeLa and LTA fibroblasts and Env.EGFP fluorescence measured at 48 h post-transfection by flow cytometry. Co-expression of miRNA targeting huPKR or huPERK increased intracellular Env.EGFP expression by 28±7% (n = 4) and 38±10% (n = 4) respectively compared with HeLa cells transfected with pNL-140.EGFP alone, or with vectors co-expressing miR-huPACT or the miR-NS control (Figure 5A). Similarly, in LTA cells the co-expression of miRNA targeting muPKR and muPERK resulted in a respective 39±14% (n = 4) and 58±15% (n = 4) increase in Env.EGFP fluorescence compared with pNL-140.EGFP alone, or vectors co-expressing miR-muPACT or the miR-NS (Figure 5B).

The secretion of the Env.EGFP fusion protein into the culture supernatants of transfected cells was measured by ELISA. In contrast with the intracellular results, in HeLa cells, the co-expression of miR-huPERK led to slightly decreased Env.EGFP secretion compared with the pNL-140.EGFP plasmid alone (Figure 5C). No change in Env.EGFP secretion was observed from any of the other miRNA co-expressing plasmids. Similarly, in transfected LTA cells, the co-expression of miR-muPACT and miR-NS decreased Env.EGFP secretion compared with the pNL-140.EGFP reporter (Figure 5D), whilst the co-expression of miR-muPKR or miR-muPERK left Env.EGFP secretion unchanged.

### Increased immunogenicity of DNA vaccines incorporating miRNA knockdown of PERK

**Figure 6:**
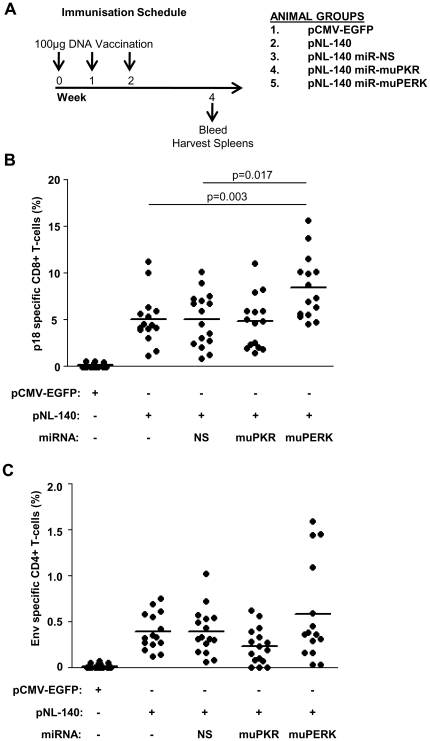
miRNA knockdown of PERK increases the immunogenicity of DNA vaccination. (**A**) Immunisation schedule. BALB/c mice were vaccinated three times at one week intervals with the indicated plasmids. Two weeks after the final vaccination, mice were sacrificed and spleens and blood samples were harvested. Splenocytes were isolated and restimulated with (**B**) 1 µM/ml p18 peptide or (**C**) recombinant NL4.3 gp140 protein. Cytokine secretion was prevented by the addition of 2 µg/ml brefeldin-A and samples were stained with monoclonal antibodies for muCD4, muCD8β, and muIFN-γ and analysed by flow cytometry. Data is shown as the proportion of CD4+ or CD8+ T-cells positive for IFN-γ secretion, minus the background stimulation observed with media alone. Pooled results are shown from two independent vaccination experiments and the mean indicated by the solid bars (n = 16 except Group 2 (pNL-140) and Group 5 (pNL-140 miR-muPERK) where n = 15). Results were compared using a Mann-Whitney U test.

In preparation for vaccine trials in mice, miRNA targeting muPKR and muPERK were sub-cloned into the DNA vaccine vector pNL-140, expressing Env gp140 from the CXCR4-tropic NL4.3 strain of HIV-1. The NL4.3 strain was selected to facilitate the ready measurement of CD8+ T-cell immunity using an MHC Class-I (H-2^d^) restricted epitope p18, previously shown to be immunodominant within the Env-specific CD8+ T cell response in BALB/c mice [40], [45]. Groups of female BALB/c mice were vaccinated with DNA plasmids expressing Env alone, or co-expressing miRNA (NS, muPKR, muPERK) and Env-specific humoral and cellular immune responses were compared to animals vaccinated with the pCMV-EGFP control plasmid (Figure 6A). After three vaccinations, no groups showed evidence of serum anti-Env antibody responses as measured by ELISA (data not shown), a finding consistent with previous DNA vaccinations using gp140 in mice [31]. We evaluated the generation of interferon gamma (IFN-γ) secreting, Env-specific CD8+ and CD4+ T-cells by intracellular cytokine secretion (ICS) assay. Following restimulation with the p18 peptide, a strong CD8+ T-cell response was detected in all groups vaccinated with Env-expressing plasmids, with responses ranging from 1–16% of total CD8+ T-cells positive for IFN-γ secretion (Figure 6B). Vaccination with the plasmid pNL-140 alone was sufficient to induce significant cellular immunity, which was unchanged by the co-expression of either miR-NS or miR-muPKR. However, vaccination with pNL-140 miR-muPERK led to a significant increase in the magnitude of CD8+ T-cell responses compared with pNL-140 miR-NS (p = 0.017) or pNL-140 alone (p = 0.003). No IFN-γ secretion was observed in the absence of peptide stimulation (data not shown), or in animals vaccinated with the control plasmid pCMV-EGFP. CD4+ cellular responses to DNA vaccination were assessed by restimulation with purified Env gp140 protein. All groups vaccinated with Env expressing DNA plasmids raised detectable but statistically equivalent CD4 T-cell responses (Figure 6C).

## Discussion

In the present study, we developed novel HIV-1 Env expression plasmids that co-expressed engineered miRNA, utilising the pri-miR-155 coding region from the human *mir155hg* gene as a scaffold. The substitution of the mature miR-155 stem with heterologous targeting sequences led to the efficient knockdown of cellular genes, indicating the terminal stem-loop required for Dicer recognition and the Drosha cleavage sites were maintained and functional. A number of miRNA expression vectors have been described based upon miRNAs such as miR-155 [46] or miR-30 [47]. More recently, vectors capable of simultaneously producing multiple miRNAs have also been described [48].

Consistent with previous studies [46], [47], we did not observe a reduction in the expression of Env when miR-155 expressing sequences were placed upstream within an artificial intron in the 5′ untranslated region (UTR), suggesting miRNA biogenesis did not lead to degradation of the Env mRNA. The cropping of intron-localised pre-miRNA by Drosha has been shown to occur co-transcriptionally but prior to intron removal [49]. The rapid kinetics of the RNAse Type III activity of Drosha allows miRNA removal, whilst the two cleaved fragments of the mRNA transcript remain tethered by components of the splicosome and with subsequent splicing catalysis occurring *in trans* [50]. Thus in the context of vaccines, the placement of miRNA expression cassettes within the intronic regions of either DNA plasmids or DNA-based viral expression vectors can facilitate the efficient *de novo* expression of miRNA effectors and antigens within a single transduced cell.

Interestingly, the co-expression of our engineered miRNA appeared to lead to an up-regulation of PKR mRNA levels, potentially indicating the engineered hairpins expressed from the miR-155-derived scaffold sequences may themselves activate a PKR response. Although PKR activation has previously been shown to be limited to dsRNA lengths greater than 30 bp [51], it is unclear if the imperfectly duplexed hairpins derived from *mir155hg*, which are greater than 30 bp in length, can act as a substrate for PKR. However, exogenous PKR expressed from the pcDNA3 plasmid did not reduce expression from the Env.EGFP reporter, indicating the normal cellular levels of PKR within HeLa cells are sufficient to limit Env expression *in vitro* and additional PKR expression induced by the expression of engineered miRNA would be unlikely to limit efficient Env expression.

In mammals, the innate intracellular immune system acts to recognise and combat viral infection, driving many common viruses to evolve protein antagonists for PKR and PERK to facilitate efficient replication and spread (Reviewed in [52]). However, the influence of innate antiviral responses on HIV-1 vaccine immunogenicity has not been extensively examined. Although HIV-1 infection is subject to cellular antiviral responses [27], [53], [54], [55], it is unclear if the expression of HIV-1 Env in the absence of viral replication can lead to activation of PKR or PERK, as previously reported for HCV E1/E2 [56] or the non-structural proteins from West Nile virus [57]. Certainly, predictive RNA-folding software such as M-fold [34] indicate the HIV-1 Env mRNA is highly structured and contains stem loops of ds-RNA as potential PKR substrates, particularly those that constitute the Rev-responsive element (RRE). Moreover, the Env glycoprotein is translated within the ER where it may be monitored by PERK. In the present study, we observed increased Env.EGFP reporter expression in the presence of a PKR dominant-negative mutant. Furthermore, the targeting of PKR or PERK, but not PACT mRNA using engineered miRNA was relatively efficient at knocking down protein expression levels and supported increased intracellular Env expression in both human and murine cell lines, suggesting the expression of Env from a native cDNA alone may be sufficient to activate intracellular antiviral surveillance leading to suboptimal expression of Env antigens.

The mechanisms underlying increased Env expression in the absence of PERK remain unclear. One possibility is the intracellular expression of Env triggers a UPR response analogous to HCV E1/E2 [56]. Thus targeting PERK expression with RNAi may prevent the downstream phosphorylation of eIF-2α, thereby limiting any inhibition of cellular or Env protein translation. Alternatively, the knockdown of PERK expression may limit the formation of heterodimers with the chaperone BiP, previously shown to guide Env folding [58], [59]. Higher concentrations of free BiP within the ER may act to sequester partially- or mis-folded Env, preventing degradation, decreasing the rate of Env turnover and thereby increasing intracellular Env accumulation. Further experiments to elucidate which of these two potential mechanisms account for the augmented Env expression seen in this study are required.

When incorporated into DNA vaccines, engineered miRNAs targeting PERK promoted significantly increased CD8+ T-cell responses in vaccinated mice, potentially indicative of increased antigen expression and MHC class-I presentation *in vivo*. Although antigen specific CD4+ T-cell responses were detected in all experimental groups vaccinated with Env, increased immunogenicity mediated by miR-PERK expression was limited to CD8+ T-cells. The observation that the knockdown of PERK failed to augment the secretion of Env proteins *in vitro* may explain the lack of augmentation of the primary CD4+ T-cell response, which is largely driven by the uptake and processing of extracellular antigens by dendritic cells, a class of professional antigen presenting cells (APC) [60], [61]. When taken together, our findings describe a model whereby APC are being directly transfected following DNA vaccination [62] and are efficiently expressing both Env and miR-muPERK *in vivo*. Reductions in intracellular PERK expression leads to increased intracellular accumulation of HIV-1 Env antigens, a proportion of which upon degradation by the proteasome and ER-resident transporter associated with antigen processing (TAP) complex [63], may facilitate increased incorporation of Env peptides into the MHC Class I presentation pathway.

In contrast with PERK, the ability of miR-PKR to increase Env expression *in vitro* did not translate into increased immunogenicity, presumably reflecting a failure to augment antigen expression or presentation *in vivo*. One possibility may be that interactions between TLR-9 and unmethylated CpG-dinucleotides within *E.coli*-derived DNA plasmids, and not HIV-1 Env expression, activates PKR responses in eukaryotic cells during transfection. Notably, the DNA vectors utilised in this study contain 15 primate-optimised CpG motifs within the backbone [64]. The stimulation of TLR-9 signalling with CpG-containing oligonucleotides (CpG-ODN) induces the secretion of type-I interferons from plasmacytoid dendritic cells [65] and monocytes [66]. Furthermore, TLR-9 is highly expressed in many tumour-derived cell lines, including HeLa cells, and the treatment of HeLa with CpG-ODN stimulates the secretion of chemokine monocyte chemoattractant protein-1 (MCP-1), indicating that TLR-9-dependent signalling pathways are functional in this cell line [67]. Alternatively, the intracellular concentrations of Env mRNA produced *in vitro* after lipid-based transfection that are available as substrate for PKR activation may be significantly higher than that obtained after plasmid uptake *in vivo* following vaccination with naked DNA plasmids. In either case, the knockdown of PKR as a molecular adjuvant may be of limited value for DNA vaccines and may have more application in recombinant viral vectors dependent upon infection and/or active replication, where intracellular concentrations of viral mRNAs may be significant.

This study provides proof-of-principle evidence that RNAi effectors incorporated into vaccine constructs can positively influence vaccine immunogenicity. Furthermore, the co-expression of engineered miRNA, or multiple miRNA, has the potential to improve the effectiveness of current vaccines that rely upon the *de novo* expression of antigens, such as DNA vaccines and recombinant viral vectors, by ameliorating confounding factors that act to limit maximal antigen expression such as the activation of cellular antiviral pathways or the induction of cellular apoptosis. Future testing of vaccine-encoded, miRNA-mediated manipulation of immune responses in a non-human primate challenge model will determine if such strategies can enhance protective efficacy.
